# Association between ankle-brachial blood pressure index and erectile dysfunction in US adults: a large population-based cross-sectional study

**DOI:** 10.3389/fendo.2024.1436043

**Published:** 2024-07-26

**Authors:** Xu Wu, Yuyang Zhang, Xuejie Zheng

**Affiliations:** ^1^ Department of Urology, First Affiliated Hospital of Anhui Medical University, Hefei, Anhui, China; ^2^ Department of Pediatrics, First Affiliated Hospital of Anhui Medical University, Hefei, Anhui, China

**Keywords:** ankle-brachial blood pressure index, erectile dysfunction, peripheral vascular disease, national health and nutrition examination survey, logistic regression

## Abstract

**Background:**

Erectile dysfunction (ED) is a very common condition among adult men and its prevalence increases with age. The ankle-brachial blood pressure index (ABPI) is a noninvasive tool used to assess peripheral vascular disease (PAD) and vascular stiffness. However, the association between ABPI and ED is unclear. We aimed to explore the association between ABPI and ED in the US population.

**Methods:**

Our study used data from two separate National Health and Nutrition Examination Survey (NHANES) datasets (2001-2002 and 2003-2004). Survey-weighted logistic regression models were used to explore the association between ABPI as a continuous variable and quartiles with ED. We further assessed the association between ABPI and ED using restricted cubic regression while selecting ABPI thresholds using two-piecewise Cox regression models. In addition, we performed subgroup analyses stratified by BMI, race, marital status, diabetes, and hypertension.

**Main outcome measure:**

ABPI was calculated by dividing the mean systolic blood pressure at the ankle by the mean systolic blood pressure at the arm.

**Results:**

Finally, 2089 participants were enrolled in this study, including 750 (35.90%) ED patients and 1339 (64.10%) participants without ED. After adjusting for all confounding covariates, logistic regression analyses showed a significant association between ABPI and ED (OR=0.19; 95% CI, 0.06-0.56, P=0.01); with ABPI as a categorical variable, compared with the lowest quartile, the OR and 95% CI for the second quartile were 0.58 (0.34-0.97; P = 0.04).Besides, splines indicated that there was an L-shaped relationship between ABPI levels and the risk of ED. Piecewise Cox regression demonstrated the inflection point at 1.14, below which the OR for ED was 0.06 (0.02-0.20; P < 0.001), and above which the OR was 2.79 (0.17-4.53; P = 0.469).

**Conclusion:**

In our study, lower ABPI was independently associated with ED risk. In addition, the lowest ABPI level associated with ED risk was 1.14, below this level, lower ABPI was associated with higher ED risk.

## Introduction

Erectile dysfunction (ED) is defined as the inability to attain or maintain a penile erection sufficient for successful vaginal intercourse (1). As a common condition, ED is prevalent in men over 40 years of age (2), and about half of men over 40 years of age are likely to have ED (3). The prevalence of ED increases gradually with age and will reach 50%-100% in men older than 70 years (4). In addition, the global prevalence of ED is estimated to reach 322 million by the year 2025 (5, 6). ED is a multifactorial disorder that can be divided into three specific categories, namely psychogenic, organic, and a mixture of both (2).

The close association between ED and cardiovascular disease (CVD) is well known (7). A number of studies have confirmed the existence of shared risk factors for ED and CVD, such as obesity, diabetes, metabolic syndrome, dyslipidemia, smoking, and sedentary lifestyle (8–10). It is widely accepted that ED is an early manifestation of CVD (11–13).

The ankle-brachial blood pressure index (ABPI) is a noninvasive tool used to assess peripheral vascular disease (PAD) and vascular stiffness (14), obtained by comparing the highest systolic blood pressure in the tibial artery with that in the brachial artery. An ABPI of < 0.9 is diagnostic of PAD (15), whereas an ABPI of > 1.3 is a reliable marker of arterial stiffness (16). First, patients with PAD are at higher risk for coronary heart disease and stroke (17), and are predictors of future cardiovascular events and mortality (18, 19). Second, arterial stiffness refers to the accumulation of plaque within the arteries, resulting in narrowing and hardening of the arteries (20), affecting multiple organs, including the brain, heart, kidneys, and lower limbs (21). Given the relationship between ED and CVD, there may be an association between ABPI and ED. In 2009, a study reported that ED was associated with PAD determined by screening ABPI testing (22). To date, no studies have explored the relationship between the overall range of ABPI and ED. However, a recent study found that the cardio-ankle vascular index of patients with ED was higher than that of healthy individuals, with no significant difference in ABPI between the two groups (23).

Is there really a correlation between ABPI and ED? Here, we conducted this study to further explore the specific association between the overall range of ABPI and ED through nationally representative data from the 2001-2004 National Health and Nutrition Examination Survey (NHANES) to provide more valuable evidence.

## Methods

### Participants

The National Health and Nutrition Examination Survey (NHANES) is a cross-sectional, stratified, multi-stage probability subgroup survey performed annually by the Centers for Disease Control and Prevention (CDC) that yields representative data (24). NHANES is used to obtain health and diet information of unstructured populations in the U.S (25). Additional details about the database have been previously reported (26). The participants in our study were collected using the NHANES database. The NHANES database is approved by the National Center for Health Statistics (NCHS) Research Ethics Review Committee, and all NHANES procedures are performed in compliance with the U.S. Department of Health and Human Services (HHS) Human Research Subjects Protection Policy. All participants provided written informed consent prior to the start of the study.

**Figure 1:**
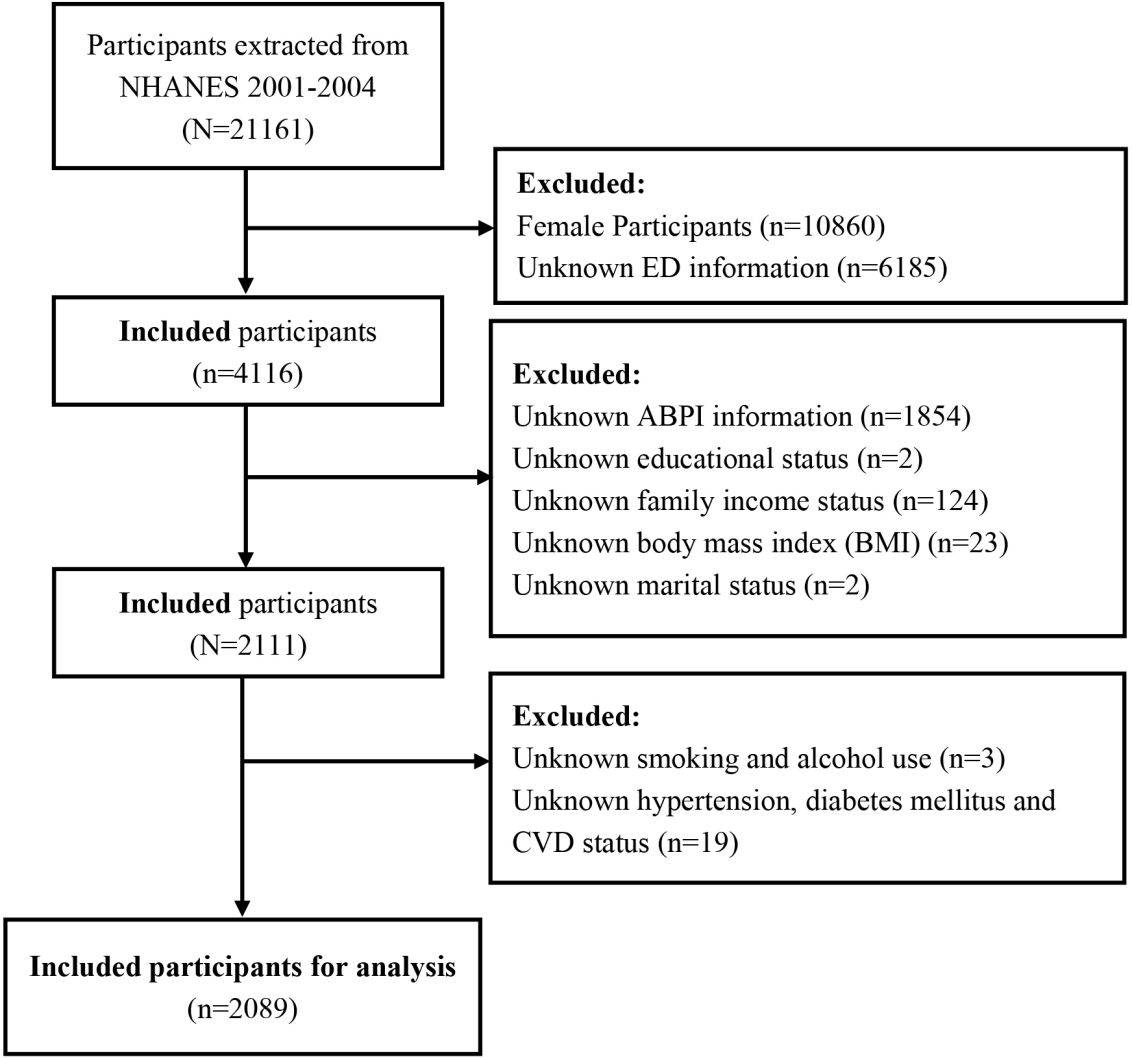
Flow chart of the study population identification from NHANES 2001 -2004.

Our study used data from two separate NHANES datasets (2001-2002 and 2003-2004) because data on ED questionnaire information was only available for these years. During these two cycles, NHANES employed rigorous and standardized data collection methods to ensure consistency and reliability across different survey cycles. Therefore, the methods for measuring ABPI and the questionnaires for assessing ED were standardized, ensuring consistency between the cycles. From 2001 to 2004, a total of 4116 males had self-reported ED information in the NHANES database. Exclusion criteria were as follows: 1. unknown ABPI information (n=1854); 2. unknown educational status (n=2); 3. unknown family income information (n=124); 4. unknown body mass index (BMI) (n=23); 5. unknown marital information (n=2); 6. unknown smoking and alcohol use (n=3); 7. unknown hypertension, diabetes mellitus and CVD status (n=19). The specific process of participant selection is shown in **Figure 1**.

### Assessment of ED

Erectile function was assessed by the following questions from the Massachusetts Male Aging Study (MMAS) (27): “How would you characterize your ability to develop and maintain an erection adequate for satisfying sexual intercourse? “ For this question, the following options are available: “never have the ability to maintain an erection,” “sometimes able to develop and maintain an erection,” “usually able to develop and maintain an erection,” “almost often or almost always able to develop and maintain an erection.” In this study, the responses “usually” and “almost often or almost always” were defined as normal erectile function, while the other two responses were defined as ED (28, 29). Moreover, the validity of the self-reported diagnostic approach to ED has been validated (30).

### Ankle-brachial blood pressure index

Blood pressure measurements were taken by trained health technicians at the mobile medical examination centers. Systolic blood pressure was measured in both arms (brachial artery) and both ankles (posterior tibial artery) of supine subjects using an automated instrument. systolic blood pressure was measured twice at each site in participants aged 40-59 years, and once at each site in participants aged 60 years or older. the ABPI was calculated by dividing the mean systolic blood pressure in the ankles by the mean systolic blood pressure in the arms (Parks Mini-Laboratory IV, Model 3100). ABPI was calculated by dividing the mean systolic blood pressure at the ankle by the mean systolic blood pressure at the arm (Parks Mini-Laboratory IV, Model 3100).

### Covariates

Confounding factors include basic characteristics: age (years), BMI, race, educational level, marital status (married or living with partner, living alone), poverty to income ratio (PIR, classified as <1.5, 1.5-3.5, and >3.5) (31). Alcohol use was categorized as (1) never alcohol use (<12 lifetime drinks); (2) former alcohol use (≥12 drinks in 1 year and no drinks in the last year, or no drinks in the last year but ≥12 lifetime drinks); (3) mild alcohol use (<2 drinks per day); (4) moderate alcohol use (≥2 drinks per day); (5) heavy alcohol use (≥3 drinks per day). The definition of smoking is when an individual smokes more than 100 cigarettes in their lifetime.

History of CVD was defined as previous coronary artery disease, angina pectoris, or heart attack; diabetes was defined as self-reported prior diagnosis of diabetes or fasting plasma glucose ≥126 mg/dL; and hypertension included systolic blood pressure ≥140 or diastolic blood pressure ≥90, or being on antihypertensive medication, or having been diagnosed with hypertension.

### Statistical methods

We performed statistical analyses using survey-weighted techniques to account for the complex sampling design of the NHANES datasets (2001-2002 and 2003-2004). By dividing the 2-year weights for each cycle by 2, we derive the new sample weights for the combined survey cycle. We used the mean ± standard error (x̅ ± SE) to describe the continuous variables, whereas categorical variables were expressed as percentage (%) ± SE. We used survey-weighted chi-square tests (for categorical variables) and survey-weighted linear regression (for continuous variables) to analyze the differences between the two groups. Weighted multivariate logistic regression models were used to explore the relationship between ABPI and ED. Three models were developed to assess the relationship between ABPI and ED: Crude model: no covariates were adjusted; Adjusted model 1: age, race, education, marital status, and PIR were adjusted; Adjusted model 2: Model 1+ BMI, alcohol intake, smoking, diabetes, CVD, and hypertension were adjusted. The strength of the correlation of the multivariate model was estimated using the ratio of ratios (OR) and 95% CI.

ABPI was converted from a continuous variable to a categorical variable based on quartiles (Q) for additional analysis. We further assessed the association between ABPI and ED using restricted cubic regression while selecting ABPI thresholds using two-piecewise Cox regression models. In addition, we performed subgroup analyses stratified by BMI, race, marital status, diabetes, and hypertension. Sensitivity analyses were conducted to verify the robustness of the findings by redefining ED using more stringent criteria. We used Empower software (www.empowerstats.com) as well as R version 4.0.2 (http://www.R-project.org, The R Foundation) to perform all statistical analyses. P < 0.05 was considered statistically significant.

## Results

### Comparison of clinical characteristics of participants with and without ED

Finally, 2089 participants were enrolled in this study, including 750 (35.90%) ED patients and 1339 (64.10%) participants without ED. **Table 1** shows the weighted distribution of baseline characteristics of the included population stratified by ED status. ABPI was significantly lower in the ED group (1.12 ± 0.01) than in the non-ED group (1.16 ± 0.00) (P<0.01). Non-ED participants (51.16 ± 0.28 years) were significantly younger than ED patients (63.60 ± 0.47) (p<0.001). Statistically significant differences in education level, PIR, and alcohol intake were found between the ED and non-ED groups (P < 0.05), and the prevalence of diabetes, CVD, and hypertension was higher in the ED group (P < 0.001).

**Table 1:** Baseline characteristics of study participants in NHANES 2001–2004, weighted.

Characteristics		History of erectile dysfunction (ED)		P-value
	Overall	No	Yes	
Number (n)	2089	1339	750	
ABPI	1.15(0.00)	1.16(0.00)	1.12(0.01)	<0.001
Age, years	54.44(0.28)	51.16(0.28)	63.60(0.47)	<0.001
Age, %				<0.001
<50	40.63(0.02)	51.06(2.00)	11.49(1.66)	
≥50	59.37(0.04)	48.94(2.00)	88.51(1.66)	
BMI, kg/m^2^	28.47(0.16)	28.27(0.21)	29.05(0.27)	0.0436
BMI, %				0.1573
BMI ≤ 25	23.02(0.02)	23.43(1.91)	21.84(1.74)	
25<BMI<30	45.07(0.03)	46.21(1.44)	41.90(2.06)	
BMI≥30	31.91(0.02)	30.36(1.86)	36.26(2.51)	
Race, %				0.268
Mexican American	4.67(0.01)	4.88(0.78)	4.08(1.36)	
Other Hispanic	3.78(0.01)	3.35(0.88)	5.00(1.81)	
Non-Hispanic White	80.39(0.06)	80.13(1.92)	81.11(2.52)	
Non-Hispanic Black	8.51(0.01)	8.75(0.89)	7.86(1.13)	
Other races	2.65(0.01)	2.90(0.74)	1.94(0.49)	
Educational level, %				<0.001
Below high school	14.65(0.01)	11.36(1.03)	23.82(2.45)	
High school	25.90(0.02)	26.92(1.25)	23.04(2.01)	
Above high school	59.46(0.03)	61.72(1.58)	53.14(2.55)	
Marital status, %				0.4258
Married or living with a partner	79.64(0.05)	79.19(1.52)	80.90(1.56)	
Living alone	20.36(0.01)	20.81(1.52)	19.10(1.56)	
PIR, %				<0.001
PIR<1.3	13.20(0.01)	12.17(0.99)	16.06(1.92)	
1.3≤PIR<3.5	31.64(0.02)	28.80(1.59)	39.59(2.50)	
PIR≥3.5	55.16(0.03)	59.03(1.95)	44.35(2.87)	
Alcohol intake, %				<0.001
Never	6.02(0.01)	5.82(1.18)	6.58(1.22)	
Former	21.94(0.02)	19.11(1.77)	29.83(2.44)	
Mild	43.90(0.03)	43.60(2.37)	44.72(2.25)	
Moderate	10.22(0.01)	11.53(1.18)	6.56(1.44)	
Heavy	17.92(0.01)	19.93(1.65)	12.31(1.88)	
Smoking, %				0.0805
No	76.45(0.04)	75.39(1.18)	79.43(2.08)	
Yes	23.55(0.02)	24.61(1.18)	20.57(2.08)	
History of diabetes, %				<0.001
No	86.00(0.04)	90.93(0.78)	72.22(1.55)	
Yes	14.00(0.01)	9.07(0.78)	27.78(1.55)	
History of CVD, %				<0.001
No	92.65(0.04)	95.42(0.59)	84.90(1.73)	
Yes	7.35(0.01)	4.58(0.59)	15.10(1.73)	
History of hypertension, %				<0.001
No	56.39(0.03)	62.53(2.19)	39.25(1.87)	
Yes	43.61(0.03)	37.47(2.19)	60.75(1.87)	

ED, erectile dysfunction; ABPI, ankle-brachial blood pressure index; BMI, body mass index; PIR, poverty income ratio; CVD, cardiovascular disease.

### Association between ABPI and ED

**Table 2** showed the association of ABPI as a continuous variable and quartiles with ED. In fully adjusted Model 2, survey-weighted logistic regression analyses showed a significant association between ABPI and ED (OR=0.19; 95% CI, 0.06-0.56, P=0.01). Similarly, with ABPI as a categorical variable, compared with the lowest quartile, the OR and 95% CI for the second quartile were 0.45 (0.30-0.69; P < 0.001) in the crude model, 0.52 (0.33-0.82, P=0.01) in the partially adjusted Model 1, and 0.58 (0.34-0.97; P = 0.04) in the fully adjusted Model 2.

**Table 2:** Weighted multivariable logistic regression for the association between ABPI and ED prevalence.

Exposure	Crude Model		Adjusted Model 1		Adjusted Model 2	
	OR (95%CI)	P value	OR (95%CI)	P value	OR (95%CI)	P value
ABPI (Continuous)	0.06(0.02,0.14)	<0.001	0.14(0.05, 0.36)	<0.001	0.19(0.06,0.56)	0.01
ABPI (Quartile)						
Q1	Ref		Ref		Ref	
Q2	0.45(0.30,0.69)	<0.001	0.52(0.33, 0.82)	0.01	**0.58(0.34,0.97)**	**0.04**
Q3	0.50(0.39,0.63)	<0.001	0.65(0.49, 0.87)	0.01	0.74(0.52,1.07)	0.10
Q4	0.47(0.31,0.70)	<0.001	0.56(0.34, 0.93)	0.03	0.62(0.34,1.12)	0.10
P for trend	0.001		0.056		0.214	

ABPI, ankle-brachial blood pressure index; ED, Erectile dysfunction; OR, odds ratio; CI, confidence interval; Q1-Q4, Quartile 1to 4; BMI, body mass index; PIR, poverty income ratio; CVD, cardiovascular disease.

**Crude Model:** no covariates were adjusted.

**Model 1:** age, race, education, marital status, and PIR were adjusted.

**Model 2:** Model 1+ BMI, alcohol intake, smoking, diabetes, CVD, and hypertension were adjusted.

The bold values provided indicate that the ABPI as quartiles in the fully adjusted model is meaningful only at Q2.

**Figure 2:**
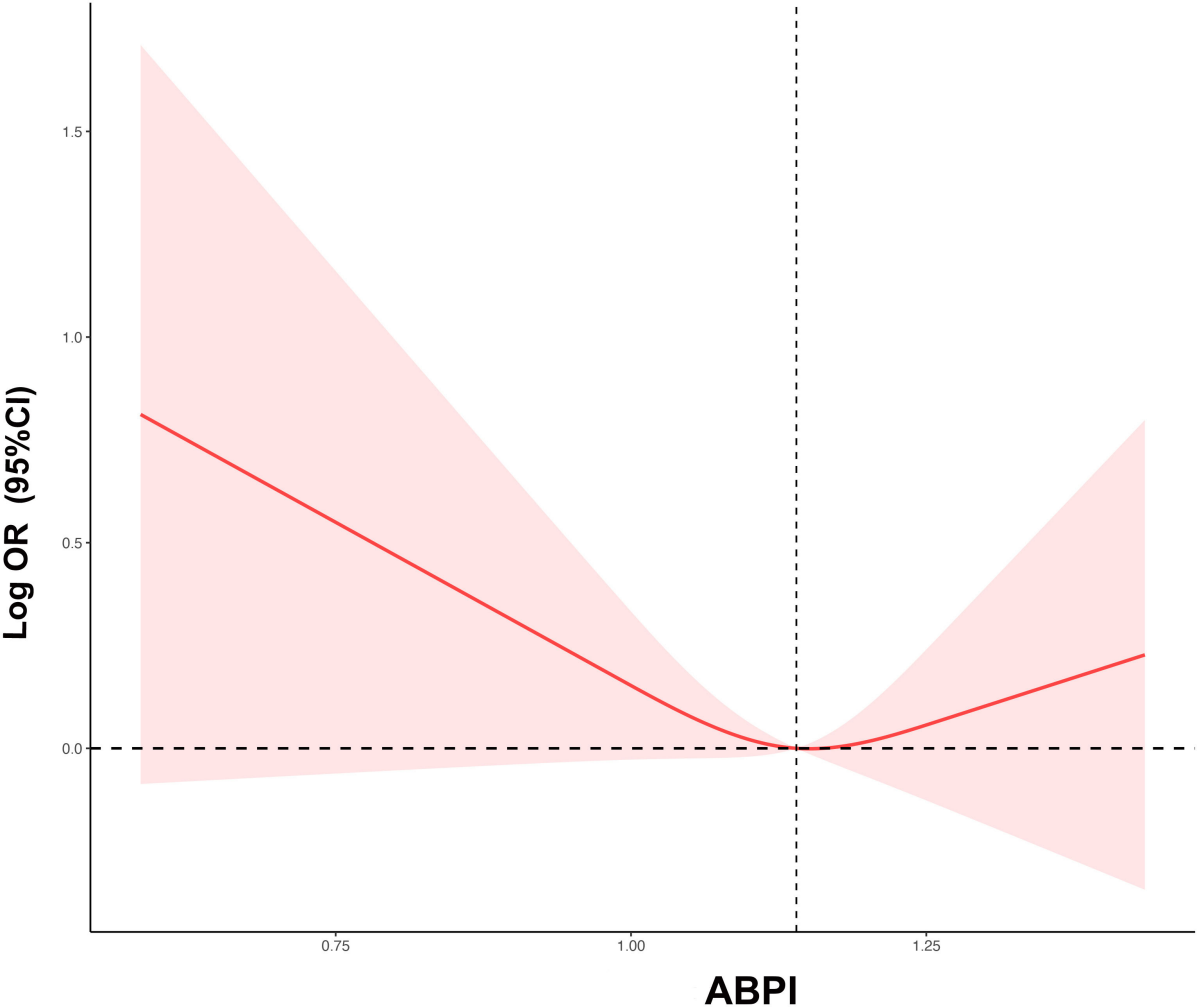
The restricted cubic regression between ABPI with ED. ABPI, ankle-brachial blood pressure index; ED, erectile dysfunction; OR, odds ratio; CI, confidence interval.

Restrictive cubic spline regression was employed to explore the dose-response relationship between ABPI and ED. The results indicated that there was an L-shaped relationship between ABPI levels and the risk of ED: as ABPI levels decreased, the risk of ED increased (**Figure 2**). Piecewise Cox regression (**Table 3**) demonstrated the inflection point at 1.14, below which the OR for ED was 0.06 (0.02-0.20; P < 0.001), and above which the OR was 2.79 (0.17-4.53; P = 0.469). The study suggested that when ABPI< 1.14, ABPI was negatively correlated with the risk of ED.

**Table 3:** Threshold Effect Analysis of Association of ABPI with ED Using Piecewise Cox Regression Models.

Outcome	OR (95%CI)	P value
Fitting model by two-piecewise linear regression Inflection point		
<1.14	0.06 (0.02,0.20)	<0.001
≥1.14	2.79 (0.17,4.53)	0.469
P for log likelihood ratio test		0.027

ABPI, ankle-brachial blood pressure index; ED, Erectile dysfunction; OR, odds ratio; CI, confidence interval.

**Adjusted Model 2:** Model 1+ BMI, alcohol intake, smoking, diabetes, CVD, and hypertension were adjusted.

### Subgroups and sensitivity analysis

**Figure 3:**
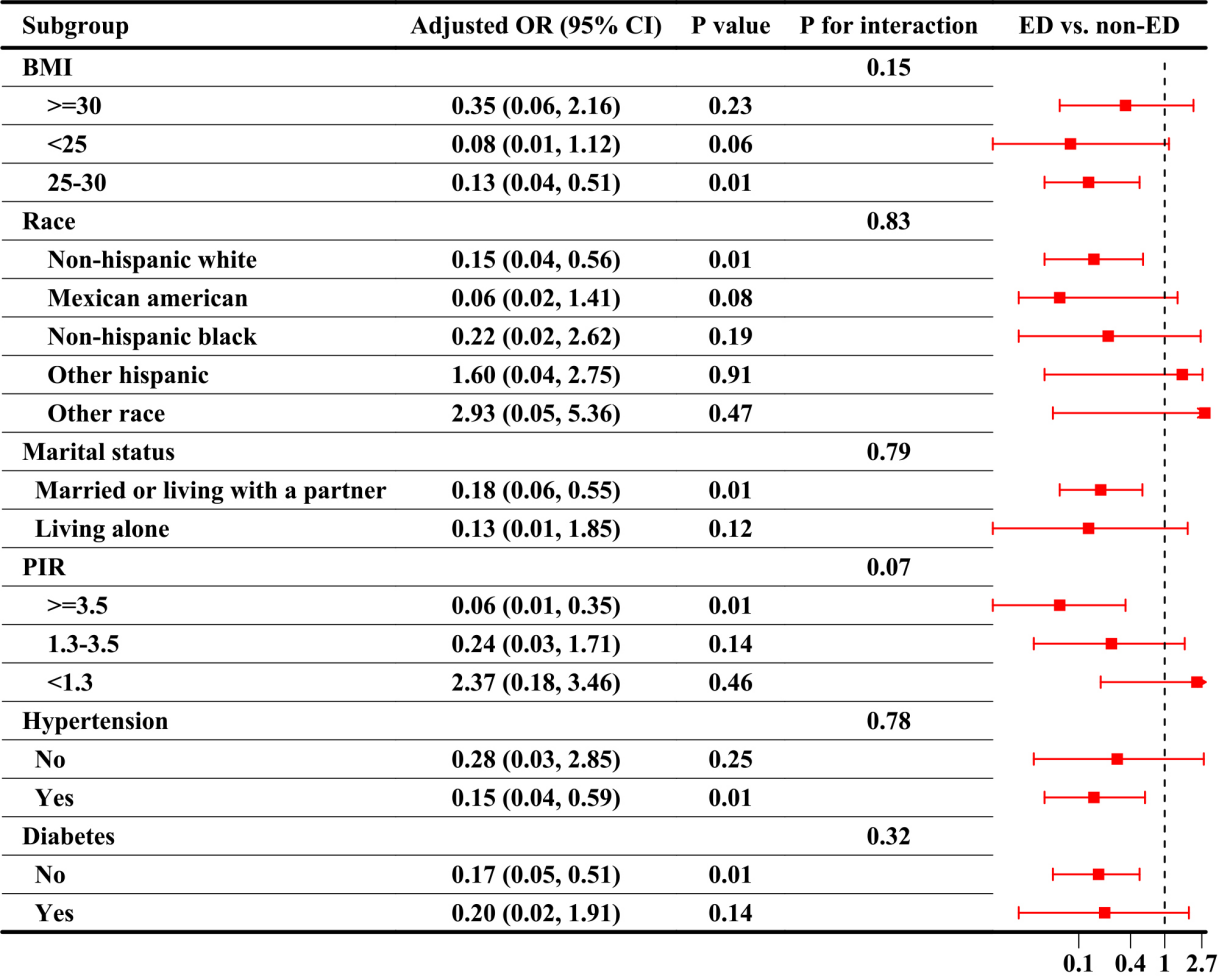
Subgroup analyses of the association between ABPI as a continuous variable and ED. ABPI, ankle-brachial blood pressure index; ED, Erectile dysfunction; OR, odds ratio, CI, confidence interval; BMI, body mass index; PIR, poverty income ratio.

Subgroup analyses were used to explore the interaction between ABPI and ED. **Figure 3** shows the results of the analysis of ABPI as a continuous variable, showing a significant association between ABPI and ED in the subgroups of BMI 25-30 (OR=0.13, 95%CI, 0.04-0.51), non-Hispanic whites (OR=0.15, 95%CI, 0.04-0.56), married or living with a partner (OR=0.18, 95%CI, 0.06-0.55), PIR ≥3.5 (OR=0.06, 95%CI, 0.01-0.35), hypertension-positive (OR=0.15, 95%CI, 0.04-0.59), and diabetes-negative (OR=0.17, 95%CI, 0.05-0.51). **Table 4** presented the results of the analysis of ABPI as quartiles, showing that the risk of ED at ABPI levels in Q2 was lower than in Q1 in the subgroups of married or living with a partner (OR=0.53, 95%CI, 0.31-0.92), PIR ≥3.5 (OR=0.42, 95%CI, 0.22-0.79), and diabetes-negative (OR=0.55, 95%CI, 0.33-0.92). There was no interaction between subgroup analyses whether ABPI was used as a continuous variable or quartiles (P for all interaction > 0.05).

**Table 4:** Subgroup analysis of the association between ABPI quartiles and ED (OR and 95%CI).

Subgroup	Quartile 1	Quartile 2	Quartile 3	Quartile 4	P for trend	P for interaction
BMI						0.25
Normal (<25 kg/m^2^)	Reference	0.54(0.25, 1.15)	1.07(0.57, 2.03)	0.78(0.34, 1.77)	0.21	
Overweight (25-30 kg/m^2^)	Reference	0.55(0.22, 1.37)	0.57(0.25, 1.32)	0.47(0.14, 1.53)	0.12	
Obese (≥30 kg/m^2^)	Reference	0.69(0.36, 1.31)	0.73(0.42, 1.25)	0.63(0.34, 1.16)	0.97	
Race						0.33
Non-Hispanic white	Reference	0.53(0.27, 1.04)	0.70(0.44, 1.10)	0.54(0.27, 1.05)	0.14	
Mexican American	Reference	0.84(0.38, 1.89)	0.98(0.38, 2.57)	0.51(0.19, 1.35)	0.24	
Non-Hispanic black	Reference	1.11(0.46, 2.71)	0.85(0.22, 3.32)	0.79(0.25, 2.52)	0.62	
Other Hispanic	Reference	0.50(0.05, 5.16)	0.93(0.08, 10.44)	1.33(0.08, 21.48)	0.84	
Other race	Reference	7.65(7.1, 8.15)	0.02(0.00,0.05)	1.55(1.29,1.86)	0.19	
Marital status						0.52
Married or living with a partner	Reference	0.53(0.31, 0.92)	0.68(0.48, 0.96)	0.61(0.34, 1.09)	0.19	
Living alone	Reference	0.70(0.27, 1.82)	0.92(0.41, 2.04)	0.50(0.17, 1.48)	0.23	
PIR						0.23
PIR≥3.5	Reference	0.42(0.22, 0.79)	0.60(0.34, 1.06)	0.41(0.22, 0.76)	0.06	
1.3≤PIR<3.5	Reference	0.52(0.24, 1.13)	0.65(0.32, 1.31)	0.71(0.29, 1.75)	0.51	
PIR<1.3	Reference	1.63(0.59, 4.47)	2.22(0.75, 6.52)	1.42(0.60, 3.36)	0.26	
Hypertension						0.75
No	Reference	0.57(0.27, 1.22)	0.70(0.38, 1.28)	0.69(0.30, 1.58)	0.55	
Yes	Reference	0.57(0.29, 1.11)	0.86(0.44, 1.66)	0.58(0.28, 1.19)	0.21	
Diabetes						0.62
No	Reference	0.55(0.33, 0.92)	0.72(0.51, 1.03)	0.65(0.38, 1.13)	0.25	
Yes	Reference	0.62(0.25, 1.55)	1.04(0.31, 3.51)	0.47(0.15, 1.49)	0.28	

ABPI, ankle-brachial blood pressure index; ED, Erectile dysfunction; OR, odds ratio; CI, confidence interval; Q1-Q4, Quartile 1to 4; BMI, body mass index; PIR, poverty income ratio; CVD, cardiovascular disease.

**Adjusted Model 2:** Model 1+ BMI, alcohol intake, smoking, diabetes, CVD, and hypertension were adjusted.

The results of the sensitivity analysis were generally consistent with the results of the main analysis (**Supplementary Table 1**). In the sensitivity analyses, we defined as ED participants who answered “never been able to get and keep an erection” to the question assessing erection, which used more stringent criteria. The results showed that there was a significant association between ABPI and ED prevalence in the fully adjusted Model 2 (Continuous variable, OR= 0.14; 95% CI, 0.05,0.42, P=0.003; Quartiles, Q2 vs Q1: OR= 0.68; 95% CI, 0.48-0.96, P=0.03).

## Discussion

This study found that the second quartile was associated with a low risk of ed in men over 40 years of age. In addition, the relationship between ABPI and ED showed an L-shaped curve, with an ABPI value of 1.14 associated with the lowest ED risk, and an ABI value below 1.14 increasing ED risk. Sensitivity analyses were consistent with the primary analysis, further determining the stability of the results. Our study is the first to assess the specific association between the overall range of the ABPI and ED through nationally representative data.

Previous studies have shown an association between lower ABPI and increased risk of malnutrition (32), diabetes (33) and CVD (34). An ABPI of < 0.9 is diagnostic of PAD (15), it is now generally accepted that an ABPI <0.9 in patients with chronic kidney disease (CKD) (35), diabetes (36), and cardio-cerebrovascular disease (37) predicts an increased risk of death. PAD is an independent predictor of mortality and morbidity due to the fact that it is usually accompanied by other atherosclerotic diseases (18, 19). In addition, a high value of ABPI > 1.3 indicates incompressible vascular calcification, reflecting arterial stiffness, which is associated with an increased risk of cardiovascular morbidity and mortality (38). Two recent NAHENS studies found that the lowest ABPI quartile in the normal range was associated with the highest risk of all-cause mortality and cardiocerebrovascular mortality, while higher ABPI were not significant (39, 40). However, there is little study on the relationship between ED and ABPI. In 2009, a study reported that ED was associated with PAD determined by screening ABPI testing (22). Consistent with that report, our findings also showed that the lowest ABI quartile was associated with risk of ED. However, a recent study found that the cardio-ankle vascular index of patients with ED was higher than that of healthy individuals, with no significant difference in ABPI between the two groups (23). The inconsistency in the findings could be attributed to the relatively small sample size of Bulbul’s study (74 ED patients, 86 healthy controls), as well as differences in the inclusion and exclusion criteria. They excluded complications such as diabetes, hypertension, CVD, and PAD with ABPI < 0.9.

However, the association between ABPI and ED yields conflicting results. This study found no significant association between ABPI higher than 1.14 and the risk of ED. ABPI exhibits an L-shaped curve relationship with ED, possibly explained by certain atherogenic mediators and inflammatory cytokines, including high-sensitivity C-reactive protein, pentraxin 3, and soluble myeloid cell expression triggering receptor-1, which decrease with increasing ABPI (41). Additionally, high ABPI is often associated with arterial calcification (42, 43) and vascular stiffness (44), counteracting protective effects. The results of the subgroup analyses indicated a lower risk of ED at the Q2 level of ABPI compared with Q1. These subgroups included patients who were married or living with a partner, had a PIR ≥3.5, and were diabetes- negative. This can be explained as follows: Marriage or a stable partnership positively influences men’s overall health and lifestyle, thereby improving their vascular health and erectile function; higher socioeconomic status is associated with better access to health resources, healthier lifestyles, and higher quality medical care; and in the absence of diabetes, maintaining good vascular health is crucial for preventing ED.

As mentioned above, lower ABPI is associated with a variety of vascular diseases and may predict atherosclerosis (18, 19, 33, 34). The relationship between ED and atherosclerotic vascular disease is closely intertwined, which may also explain the association between lower ABPI and ED risk. Nitric oxide (NO) can mediate various anti-atherosclerotic properties, including effects on inflammation, platelet aggregation, and smooth muscle proliferation, and impaired NO levels are an early finding in atherosclerosis (45). Normal erectile function is particularly sensitive to reduce NO, and ED may be an early clinical manifestation of underlying vascular disease and NO deficiency (46). Additionally, penile arteries are relatively small, and with the progression of occlusive diseases, clinical manifestations may occur earlier in the penile vascular bed than in other vascular beds (47). In summary, lower ABPI predicts a possible risk of atherosclerotic lesions in the lower limb arteries, and ED symptoms may already be present at this stage. As Polonsky et al. suggest, ED may serve as an independent predictor of occult PAD identified through prospective ABPI testing (22). In addition, chronic inflammation is a common underlying pathology in both PAD and ED. Inflammatory cytokines such as C-reactive protein (CRP) and interleukins are elevated in patients with atherosclerosis and endothelial dysfunction. These inflammatory mediators contribute to the progression of vascular disease and directly affect erectile function by inducing vascular damage and impairing smooth muscle relaxation (45). The neurovascular interplay is crucial for erectile function. In conditions with compromised ABPI, there is often concurrent neurovascular dysfunction. The impaired neural regulation of blood flow, combined with vascular insufficiency, disrupts the normal erectile process (22, 47).

The present study has some limitations. First, this study was cross-sectional and could not provide a causal relationship between ABPI and ED. Second, due to the limitations of the NAHENS data, we were only able to study men in specific age groups. Third, the cross-sectional nature of our study captures ABPI at a single point in time, which may not fully reflect the dynamic nature of vascular health. As a result, the observed associations between ABPI and erectile dysfunction (ED) might be influenced by these temporal variations. Longitudinal studies that track changes in ABPI and ED over time are needed to provide a more comprehensive understanding of the relationship. Additionally, although we adjusted for many confounding factors, there may still be residual confounding factors due to data limitations, such as lifestyle interventions and the use of certain medications (antihypertensives, lipid-lowering drugs, and antidepressants). Finally, while the MMAS questionnaire is a validated tool for assessing erectile function (30), it may have limitations compared to the more widely used International Index of Erectile Function (IIEF). The IIEF provides a more comprehensive assessment of erectile function, including domains such as orgasmic function, sexual desire, and overall satisfaction. The use of MMAS in this study, although validated, may not capture the full spectrum of ED symptoms as effectively as the IIEF.

However, our study could provide more detailed suggestions for future research. Future research should focus on longitudinal studies to establish a causal relationship between ABPI and ED. Tracking changes in ABPI and erectile function over time could provide valuable information on the progression and potential reversibility of vascular contributions to ED. Moreover, detailed mechanistic studies are needed to explore the specific biological pathways linking ABPI with ED. Investigating the roles of endothelial function, NO synthesis, and inflammation in larger, diverse populations could yield critical insights into the underlying mechanisms. Finally, clinical trials examining the impact of interventions targeting vascular health on erectile function are essential. Studies assessing the effects of lifestyle modifications, pharmacological treatments, or surgical interventions on both ABPI and ED outcomes could inform effective management strategies for patients with coexisting vascular diseases and ED.

## Conclusion

In our study, lower ABPI was independently associated with ED risk. In addition, the lowest ABPI level associated with ED risk was 1.14, below this level, lower ABPI was associated with higher ED risk. This suggests that clinicians may consider assessing ABPI in individuals with ED and evaluating erectile function in those with lower ABPI levels. Clinicians should consider incorporating ABPI measurements into routine assessments, especially for patients with CVD risk factors. Early detection of vascular impairment can prompt timely interventions to prevent the progression of ED. Additionally, understanding the relationship between ABPI and ED can help in developing personalized treatment plans. Future studies should conduct longitudinal investigations to determine causality, as well as interventional studies to assess whether treatment of peripheral vascular disease improves ED.

## Data availability statement

The original contributions presented in the study are included in the article/**Supplementary Material**. Further inquiries can be directed to the corresponding author.

## Ethics statement

The NHANES database is open to the public and therefore the ethical review of this study was exempt. All participants provided written informed consent prior to the start of the study.

## Author contributions

XW: Conceptualization, Data curation, Formal Analysis, Investigation, Methodology, Project administration, Resources, Software, Supervision, Validation, Visualization, Writing – original draft, Writing – review & editing. YZ: Methodology, Software, Supervision, Validation, Writing – review & editing. XZ: Conceptualization, Data curation, Formal Analysis, Funding acquisition, Project administration, Resources, Software, Writing – review & editing.

## References

[1] Consensus development conference statement. National Institutes of Health. Impotence. December 7-9, 1992. Int J Impot Res. (1993) 5:181–284. 8173631

[2] Shamloul R Ghanem H. Erectile dysfunction. Lancet. (2013) 381:153–65. doi: 10.1016/S0140-6736(12)60520-0 23040455

[3] Najari BB Kashanian JA. Erectile dysfunction. JAMA. (2016) 316:1838. doi: 10.1001/jama.2016.12284 27802547

[4] Maiorino MI Bellastella G Esposito K. Diabetes and sexual dysfunction: current perspectives. Diabetes Metab Syndr Obes. (2014) 7:95–105. doi: 10.2147/DMSO 24623985 PMC3949699

[5] Ayta IA McKinlay JB Krane RJ. The likely worldwide increase in erectile dysfunction between 1995 and 2025 and some possible policy consequences. BJU Int. (1999) 84:50–6. doi: 10.1046/j.1464-410x.1999.00142.x 10444124

[6] Bacon CG Mittleman MA Kawachi I Giovannucci E Glasser DB Rimm EB. Sexual function in men older than 50 years of age: results from the health professionals follow-up study. Ann Intern Med. (2003) 139:161–8. doi: 10.7326/0003-4819-139-3-200308050-00005 12899583

[7] Gandaglia G Briganti A Jackson G Kloner RA Montorsi F Montorsi P. A systematic review of the association between erectile dysfunction and cardiovascular disease. Eur Urol. (2014) 65:968–78. doi: 10.1016/j.eururo.2013.08.023 24011423

[8] Buvat J Maggi M Gooren L Guay AT Kaufman J Morgentaler A. Endocrine aspects of male sexual dysfunctions. J Sex Med. (2010) 7:1627–56. doi: 10.1111/j.1743-6109.2010.01780.x 20388162

[9] Jackson G Montorsi P Adams MA Anis T El-Sakka A Miner M. Cardiovascular aspects of sexual medicine. J Sex Med. (2010) 7:1608–26. doi: 10.1111/j.1743-6109.2010.01779.x 20388161

[10] Salonia A Castagna G Saccà A Ferrari M Capitanio U Castiglione F. Is erectile dysfunction a reliable proxy of general male health status? The case for the International Index of Erectile Function-Erectile Function domain. J Sex Med. (2012) 9:2708–15. doi: 10.1111/j.1743-6109.2012.02869.x 22897643

[11] Clark NG Fox KM Grandy S. Symptoms of diabetes and their association with the risk and presence of diabetes: findings from the Study to Help Improve Early evaluation and management of risk factors Leading to Diabetes (SHIELD). Diabetes Care. (2007) 30:2868–73. doi: 10.2337/dc07-0816 17712027

[12] Inman BA Sauver JL Jacobson DJ McGree ME Nehra A Lieber MM. A population-based, longitudinal study of erectile dysfunction and future coronary artery disease. Mayo Clin Proc. (2009) 84:108–13. doi: 10.4065/84.2.108 PMC2664580 19181643

[13] Chung SD Chen YK Lin HC Lin HC. Increased risk of stroke among men with erectile dysfunction: a nationwide population-based study. J Sex Med. (2011) 8:240–6. doi: 10.1111/j.1743-6109.2010.01973.x 20722781

[14] Watson EL Patel B Katsogridakis E Pepper CJ Messeder SJ Saratzis A. Selecting portable ankle/toe brachial pressure index systems for a peripheral arterial disease population screening programme: a systematic review, clinical evaluation exercise, and consensus process. Eur J Vasc Endovasc Surg. (2022) 64:693–702. doi: 10.1016/j.ejvs.2022.08.008 35970334

[15] Formosa C Cassar K Gatt A Mizzi A Mizzi S Camileri KP. Hidden dangers revealed by misdiagnosed peripheral arterial disease using ABPI measurement. Diabetes Res Clin Pract. (2013) 102:112–6. doi: 10.1016/j.diabres.2013.10.006 24209599

[16] Kendrick J Ix JH Targher G Smits G Chonchol M. Relation of serum phosphorus levels to ankle brachial pressure index (from the Third National Health and Nutrition Examination Survey). Am J Cardiol. (2010) 106:564–8. doi: 10.1016/j.amjcard.2010.03.070 PMC2943573 20691317

[17] Grenon SM Hiramoto J Smolderen KG Vittinghoff E Whooley MA Cohen BE. Association between depression and peripheral artery disease: insights from the heart and soul study. J Am Heart Assoc. (2012) 1:e002667. doi: 10.1161/jaha.112.002667 23130170 PMC3487348

[18] Aboyans V Ho E Denenberg JO Ho LA Natarajan L Criqui MH. The association between elevated ankle systolic pressures and peripheral occlusive arterial disease in diabetic and nondiabetic subjects. J Vasc Surg. (2008) 48:1197–203. doi: 10.1016/j.jvs.2008.06.005 18692981

[19] Criqui MH McClelland RL McDermott MM Allison MA Blumenthal RS Aboyans V. The ankle-brachial index and incident cardiovascular events in the MESA (Multi-Ethnic Study of Atherosclerosis). J Am Coll Cardiol. (2010) 56:1506–12. doi: 10.1016/j.jacc.2010.04.060 PMC2962558 20951328

[20] Lacolley P Regnault V Segers P Laurent S. Vascular smooth muscle cells and arterial stiffening: relevance in development, aging, and disease. Physiol Rev. (2017) 97:1555–617. doi: 10.1152/physrev.00003.2017 28954852

[21] Laurent S Boutouyrie P. Arterial stiffness and hypertension in the elderly. Front Cardiovasc Med. (2020) 7:544302. doi: 10.3389/fcvm.2020.544302 33330638 PMC7673379

[22] Polonsky TS Taillon LA Sheth H Min JK Archer SL Ward RP. The association between erectile dysfunction and peripheral arterial disease as determined by screening ankle-brachial index testing. Atherosclerosis. (2009) 207:440–4. doi: 10.1016/j.atherosclerosis.2009.05.005 19501825

[23] Bulbul E Aydin E Yilmaz E. Evaluation of endothelial dysfunction with cardio-ankle vascular index measurements in patients with erectile dysfunction. Andrology. (2022) 10:926–30. doi: 10.1111/andr.13191 35466575

[24] Mao W Hu Q Chen S Chen Y Luo M Zhang Z. Polyfluoroalkyl chemicals and the risk of kidney stones in US adults: A population-based study. Ecotoxicol Environ Saf. (2021) 208:111497. doi: 10.1016/j.ecoenv.2020.111497 33091773

[25] Mao W Wu J Zhang Z Xu Z Xu B Chen M. Neutrophil-lymphocyte ratio acts as a novel diagnostic biomarker for kidney stone prevalence and number of stones passed. Transl Androl Urol. (2021) 10:77–86. doi: 10.21037/tau-20-890 33532298 PMC7844488

[26] Curtin LR Mohadjer LK Dohrmann SM Kruszon-Moran D Mirel LB Carroll MD. National Health and Nutrition Examination Survey: sample design 2007-2010. Vital Health Stat. (2013) 2:1–23. 25090039

[27] Derby CA Araujo AB Johannes CB Feldman HA McKinlay JB. Measurement of erectile dysfunction in population-based studies: the use of a single question self-assessment in the Massachusetts Male Aging Study. Int J Impot Res. (2000) 12:197–204. doi: 10.1038/sj.ijir.3900542 11079360

[28] Lopez DS Wang R Tsilidis KK Zhu H Daniel CR Sinha A. Role of caffeine intake on erectile dysfunction in US men: results from NHANES 2001-2004. PloS One. (2014) 10:e0123547. doi: 10.1371/journal.pone.0123547 25919661 PMC4412629

[29] Farag YMK Guallar E Zhao D Kalyani RR Blaha MJ Feldman DI. Vitamin D deficiency is independently associated with greater prevalence of erectile dysfunction: The National Health and Nutrition Examination Survey (NHANES) 2001-2004. Atherosclerosis. (2016) 252:61–7. doi: 10.1016/j.atherosclerosis.2016.07.921 PMC5035618 27505344

[30] O'Donnell AB Araujo AB Goldstein I McKinlay JB. The validity of a single-question self-report of erectile dysfunction. Results from the Massachusetts Male Aging Study. J Gen Intern Med. (2005) 20:515–9. doi: 10.1111/j.1525-1497.2005.0076.x PMC1490137 15987326

[31] Rahman HH Niemann D Munson-McGee SH. Association of albumin to creatinine ratio with urinary arsenic and metal exposure: evidence from NHANES 2015-2016. Int Urol Nephrol. (2022) 54:1343–53. doi: 10.1007/s11255-021-03018-y 34643861

[32] MuzemboBANaganoYDumavibhatNNgatuNRMatsuiTBhattiSA. Ankle-brachial pressure index and mini nutritional assessment in community-dwelling elderly people. J Nutr Health Aging. (2013) 17:370–6. doi: 10.1007/s12603-012-0412-6 23538661

[33] Jensen SA Vatten LJ Myhre HO. The association between diabetes mellitus and the prevalence of intermittent claudication: the HUNT study. Vasc Med. (2008) 13:239–44. doi: 10.1177/1358863x08094800 18940899

[34] Filippella M Lillaz E Ciccarelli A Giardina S Massimetti E Navaretta F. Ankle brachial pressure index usefulness as predictor factor for coronary heart disease in diabetic patients. J Endocrinol Invest. (2007) 30:721–5. doi: 10.1007/bf03350808 17993762

[35] Chen HY Wei F Wang LH Wang Z Meng J Yu HB. Abnormal ankle-brachial index and risk of cardiovascular or all-cause mortality in patients with chronic kidney disease: a meta-analysis. J Nephrol. (2017) 30:493–501. doi: 10.1007/s40620-017-0376-z 28197971

[36] Hanssen NM Huijberts MS Schalkwijk CG Nijpels G Dekker JM Stehouwer CD. Associations between the ankle-brachial index and cardiovascular and all-cause mortality are similar in individuals without and with type 2 diabetes: nineteen-year follow-up of a population-based cohort study. Diabetes Care. (2012) 35:1731–5. doi: 10.2337/dc12-0178 PMC3402264 22699294

[37] Liu L Sun H Nie F Hu X. Prognostic value of abnormal ankle-brachial index in patients with coronary artery disease: A meta-analysis. Angiology. (2020) 71:491–7. doi: 10.1177/0003319720911582 32166959

[38] Criqui MH Aboyans V. Epidemiology of peripheral artery disease. Circ Res. (2015) 116:1509–26. doi: 10.1161/circresaha.116.303849 25908725

[39] Meng Z Jiang Y Xu C Zheng H Li H. Association between ankle-brachial blood pressure index with all-cause and cardiovascular mortality in adults without arterial stiffness. BMC Geriatr. (2023) 23:635. doi: 10.1186/s12877-023-04332-z 37814212 PMC10563285

[40] Xu C Tian Q Yu H Ge W Zheng H Huang D. Predictive value of the ankle-brachial index for all-cause and cardio-cerebrovascular mortality. Angiology. (2023) 74:649–56. doi: 10.1177/00033197221121016 36052942

[41] Ozkaramanli Gur D Gur O Guzel S Akyuz A Gurkan S Alpsoy S. Inflammatory mediators across the spectrum of ankle-brachial index. J Atheroscler Thromb. (2019) 26:351–61. doi: 10.5551/jat.44891 PMC6456454 30249941

[42] Allison MA Laughlin GA Barrett-Connor E Langer R. Association between the ankle-brachial index and future coronary calcium (the Rancho Bernardo study). Am J Cardiol. (2006) 97:181–6. doi: 10.1016/j.amjcard.2005.08.019 16442359

[43] Adragao T Pires A Branco P Castro R Oliveira A Nogueira C. Ankle–brachial index, vascular calcifications and mortality in dialysis patients. Nephrol Dial Transplant. (2012) 27:318–25. doi: 10.1093/ndt/gfr233 21551082

[44] Aboyans V Criqui MH Abraham P Allison MA Creager MA Diehm C. Measurement and interpretation of the ankle-brachial index: a scientific statement from the American Heart Association. Circulation. (2012) 126:2890–909. doi: 10.1161/CIR.0b013e318276fbcb 23159553

[45] Davignon J Ganz P. Role of endothelial dysfunction in atherosclerosis. Circulation. (2004) 109:Iii27–32. doi: 10.1161/01.CIR.0000131515.03336.f8 15198963

[46] Bush PA Aronson WJ Buga GM Rajfer J Ignarro LJ. Nitric oxide is a potent relaxant of human and rabbit corpus cavernosum. J Urol. (1992) 147:1650–5. doi: 10.1016/s0022-5347(17)37671-1 1317469

[47] Montorsi P Montorsi F Schulman CC. Is erectile dysfunction the "tip of the iceberg" of a systemic vascular disorder? Eur Urol. (2003) 44:352–4. doi: 10.1016/s0302-2838(03)00307-5 12932935

