# Supplementary material for: Association between ankle-brachial blood pressure index and erectile dysfunction in US adults: a large population-based cross-sectional study
Source: Front Endocrinol (Lausanne). 2024 Jul 26;15:1436043. doi: 10.3389/fendo.2024.1436043 (PMC11310141; doi:10.3389/fendo.2024.1436043)
Supplement: Supplementary file 1 [file Table_1.docx]

**Table S1.** Sensitivity analysis for the association between ABPI and ED (stringent criteria), weighted.

| Exposure | Model 2 | |
| --- | --- | --- |
|  | OR (95%CI) | P value |
| ABPI (Continuous) | 0.14(0.05,0.42) | 0.003 |
| ABPI (Quartile) |  |  |
| Q1 | Ref |  |
| Q2 | 0.68(0.48,0.96) | 0.03 |
| Q3 | 0.82(0.54,1.26) | 0.33 |
| Q4 | 0.78(0.54,1.15) | 0.19 |

**Abbreviations:** ABPI, ankle-brachial blood pressure index; ED, Erectile dysfunction; OR, odds ratio; CI, confidence interval, Q1-Q4: Quartile 1to 4; BMI, body mass index; PIR, poverty income ratio; CVD, cardiovascular disease.

**Adjusted Model 2:** Model 1+ BMI, alcohol intake, smoking, diabetes, CVD, and hypertension were adjusted.
